# Interventions to Address the Health and Well-Being of Married Adolescents: A Systematic Review

**DOI:** 10.9745/GHSP-D-23-00425

**Published:** 2024-08-27

**Authors:** Manahil Siddiqi, Margaret E. Greene, Alexandra Stoppel, Charles Allegar

**Affiliations:** a Department of Health Systems and Population Health, University of Washington School of Public Health, Seattle, WA, USA.; b GreeneWorks, Washington, DC, USA.; c Independent consultant, Ballwin, MO, USA.; d Independent consultant, Washington, DC, USA.

## Abstract

Child marriage, defined by the United Nations as marriage before the age of 18 years, is a widespread practice with serious health and social consequences. We systematically reviewed academic and gray literature to learn what is known about the current state of the evidence for interventions to respond to the needs of married adolescents. Our analysis of the 29 included intervention studies identified variability across sectors, with most programs focusing on sexual and reproductive health (SRH) and maternal health. We found that interventions occur mostly at a small scale, are geographically concentrated, and focus on SRH to the neglect of other sectors. There is a need for programs to focus on improving educational and economic outcomes among married girls, as well as increased efforts to support equitable marital relations and engage men and boys. The emphasis on preventing child marriage must be matched by efforts to mitigate and respond to the practice.

## Abstract (precis)

This review concludes that little research and programmatic attention is paid to the needs and vulnerabilities of married girls as if it were too late to reach them, and limited effort is made to address relationship dynamics and other conditions within marriage other than sexual and reproductive health.

## Teaser

**Key Findings:**

- Despite awareness of the harmful health and social consequences of child marriage, married girls and boys remain an overlooked population in programming.
- The limited interventions targeting married adolescents are geographically concentrated in sub-Saharan Africa and South Asia, implemented at a small scale, focus on sexual and reproductive health or maternal health to the neglect of other areas, including voice and agency, violence prevention, economic livelihoods, schooling, and rights and access to legal support.
- There is a lack of programmatic attention to boys and men in the context of child marriage response.

**Key Implications:**

- Program designers and implementers must identify and address the needs of married adolescents through targeted evidence-based interventions that encompass timely identification and referral of adolescents affected by child marriage; collaboration between child protection, education and social protection sectors; and employ evidence-based design and delivery.
- Policymakers should invest in and scale up programs and services that not only prevent child marriage but also mitigate the harmful consequences faced by survivors of child marriage, particularly girls who remain disproportionately affected by the harmful effects of child marriage.

## INTRODUCTION

Globally, 650 million girls and 115 million boys are affected by child marriage. Child marriage disproportionately affects girls, with 1 in 5 young women aged 20 to 24 years old married before 18, compared to 1 in 30 young men.^1^ The practice constitutes a grave threat to a person’s health, well-being, and future prospects. In the past 20 years, our understanding of the effects of child marriage, particularly among girls, has expanded. Married girls younger than 15 years are more likely to experience maternal and neonatal complications and die in childbirth.^2^ A review of the mental health consequences of child marriage revealed depression, anxiety, and suicidal thoughts and attempts, as well as emotional distress related to poverty, intimate partner violence, challenges in childbirth, isolation, and loss of autonomy, were all associated with child marriage.^3^ A recent study examining child marriage and the risk of hypertension found that marrying before the age of 18 years was significantly associated with an almost two-fold increase in the risk of hypertension among young adult women.^4^ Vulnerability to health and social risks can be greater the younger girls are married. For example, a study among Ethiopian women found that girls married very early (before age 16 years) reported more partner assaults and sexual coercion than girls who married later.^5^

In addition to the effects of child marriage on health and safety, child marriage hinders life choices. The termination of girls’ schooling is closely associated with child marriage. Married girls face practical barriers to continuing their education, including responsibilities to take care of the home, extended family, and children, in addition to a lack of support for re-enrolment in school and stigma. Depriving girls of schooling limits prospects for future earnings and productivity and hinders girls’ overall decision-making power within the household, community, and broader society.^6^ For both girls and boys faced with pressure to provide for their families, child marriage may increase the risk of child labor. Research shows that boys experience pressure to marry as soon as they achieve economic independence and appear ready to support a family.^7^ Where employment is limited, returns to schooling are low, poverty is widespread, and more boys are driven out of school and into the labor force and early marriage.

Despite awareness of these consequences, married girls and boys remain overlooked. The 2030 Agenda for Sustainable Development includes a specific target for eliminating child marriage and includes goals related to quality education, gender inequality, and good health and well-being.^8^ Yet the Sustainable Development Goals include no explicit reference to addressing the needs of married adolescents. Limited attention to the needs and vulnerabilities of married girls has historically led to limited resources and programming tailored for this population. A systematic scoping review of the child marriage evidence over the last 2 decades concluded that while ample evidence exists documenting the problem of child marriage, including its prevalence and trends, drivers, and consequences, a shift must occur from diagnosis to response.^9^ As the world awakens to the damaging and lasting consequences of child marriage, it is vital we identify the unique needs of those affected by this practice and how to respond to and support this population.

It is vital we identify the unique needs of those affected by child marriage and how to respond to and support this population.

This systematic review aimed to assess the evidence on interventions that target married adolescents. Previous reviews of child marriage research have focused more narrowly on interventions to prevent child marriage.^10^^–^^13^ To date, there has been no published review on interventions to address the needs of married adolescents. In this review, the term “married adolescents” refers to all individuals married before the age of 18 years, the majority of whom are girls. This review aims to answer the following research questions: How are the needs of married adolescents addressed by interventions? What characteristics distinguish programs serving adolescents and young adults married as children and what can we learn from effective interventions? Based on our findings, we present a range of recommendations for practice and future research.

## METHODS

### Search Strategy and Selection Criteria

The study design follows the Preferred Reporting Items for Systematic Reviews and Meta-Analysis (PRISMA) guidelines (Table 1). This systematic review builds on a comprehensive scoping review of child marriage studies, published between 2000 and 2019, which was registered with Open Science Framework (https://osf.io/awh8v). The full protocol was published in 2019, and the review was published in 2020.^9^^,^^14^ The scoping review was designed as a precursor for a series of systematic reviews that address specific questions related to interventions focusing on child marriage. From the preliminary scoping review, we focus for this review only on those studies that reported on interventions and included participants that were married or in union before the age of 18 years. Building on this initial review, we additionally searched 6 databases to include literature from January 2020 to January 2022. Research published before 2000 was not included in this review because child marriage was not commonly recognized as a human rights issue before 2000, and consequently, the issue received very limited research and programmatic attention. As this is a systematic review of published literature, ethics approval was not sought.

**TABLE 1.** Inclusion and Exclusion Criteria for Systematic Review of Interventions to Address Married Adolescents’ Needs

**Category**	**Included**	**Excluded**
Study types and design	Impact evaluations of programming using experimental or quasi-experimental designs (including mixed methods approaches)	Publications that describe process evaluations, use only qualitative methods, use nonexperimental research designs, or theoretical or conceptual articles
Focus area	Interventions that seek to improve health and/or social outcomes for individuals married as children—study sample must have included individuals married or in union before the age of 18 years	Publications that include only unmarried individuals
Publication type	Peer-reviewed journal articles and project reports	Policy briefs, programmatic literature that does not describe study results and effectiveness in detail
Publication date	January 2000 to January 2022	Documents published before 2000
Language	English	Languages other than English

**FIGURE 1:**
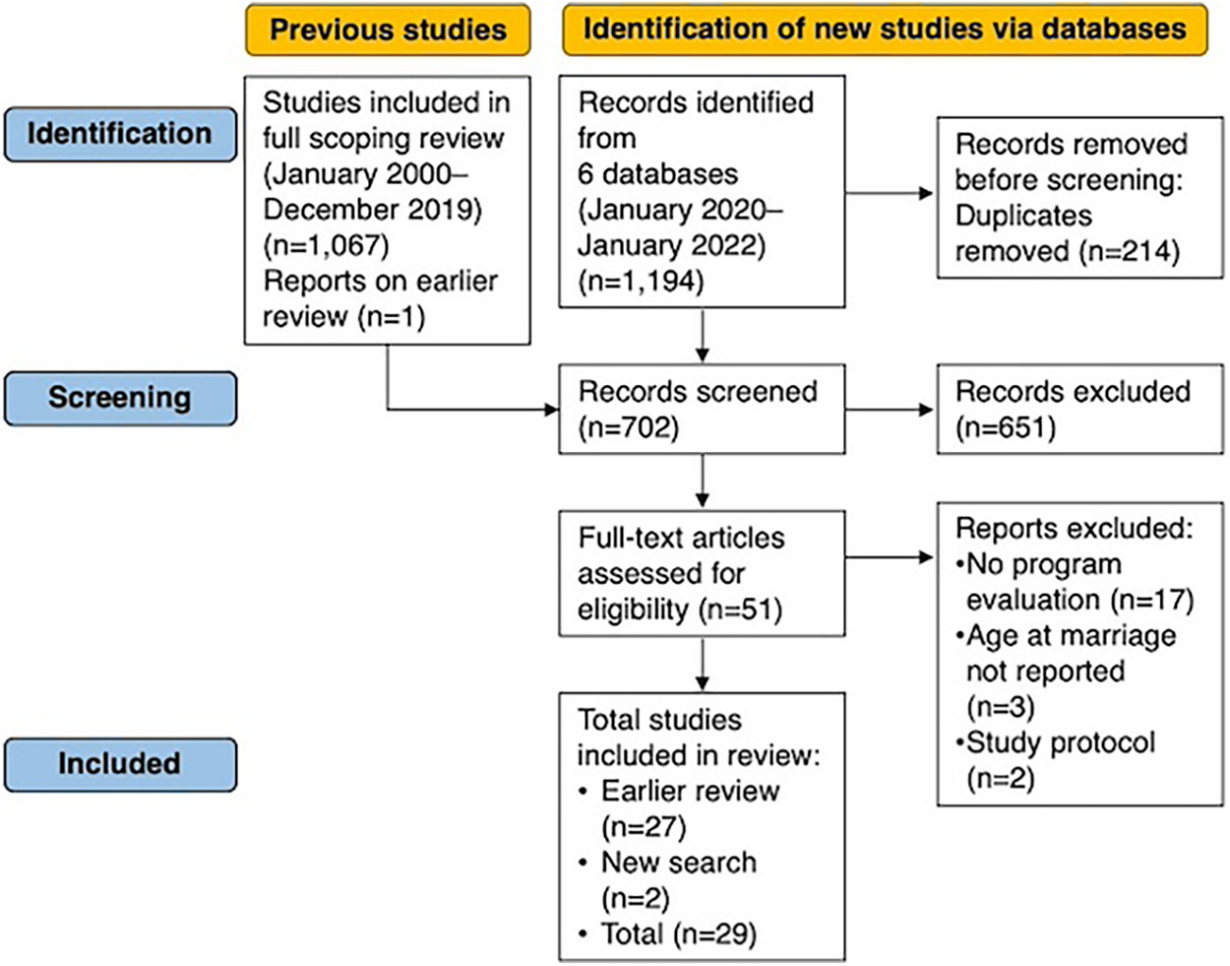
Flow Chart of Studies Included in Review of Interventions to Respond to Needs of Married Adolescents

The broader initial review identified a total of 1,067 publications, which were then screened to include only those that were interventions and included participants that were married or in union before the age of 18 years. We searched the following 6 databases to include literature from January 2020 to January 2022: PubMed, PsychINFO, Embase, CINHAL Plus, Web of Science, and the Cochrane Library. Search terms included variations of terms such as “child brides,” “child wives,” “married adolescents,” “child marriage,” and “early marriage.” We consulted with an informational specialist to develop a search strategy adapted for each database. A title and abstract screening of studies from the previous review (2000–2019) and the records identified from the 6 databases (2020–2022) resulted in 702 studies screened (Figure 1).

### Inclusion Criteria and Study Selection

The titles and abstracts of 702 papers were reviewed by 2 authors (MS and MG) independently to assess suitability for inclusion. Table 1 documents our inclusion criteria and examples for exclusion. We included titles and/or abstracts describing interventions that included in their program individuals married or in union before the age of 18 years. This yielded 51 publications. During the full-text screenings, data were collected from each report (by MS, CA, and AS) into a Microsoft Excel spreadsheet, and the full team (AS, CA, MS, and MG) reviewed, discussed, and finalized the data extraction and eligibility for inclusion. We excluded publications not associated with program evaluations. We also excluded publications in which age at marriage was not reported.

### Data Extraction

In total, 29 articles met the eligibility criteria for this systematic review. We charted the data using a data extraction form to document multiple study characteristics (i.e., location, duration, target population, setting, sample size, evaluation design, intervention objectives, components, and results).

### Quality Assessment and Data Synthesis

We adapted a risk of bias tool to conduct a quality assessment of the studies based on key characteristics (study design, attrition, sample size, selection bias, measurement of exposure, outcome measurement, and clarity of reporting) and assessment of methodological quality.^7^ This assessment of bias was conducted independently by 2 authors (MS and CA), followed by a joint discussion with the study team (MS, CA, MG, LA). Studies were given a score that classified them into low, medium, or high-quality.

**FIGURE 2:**
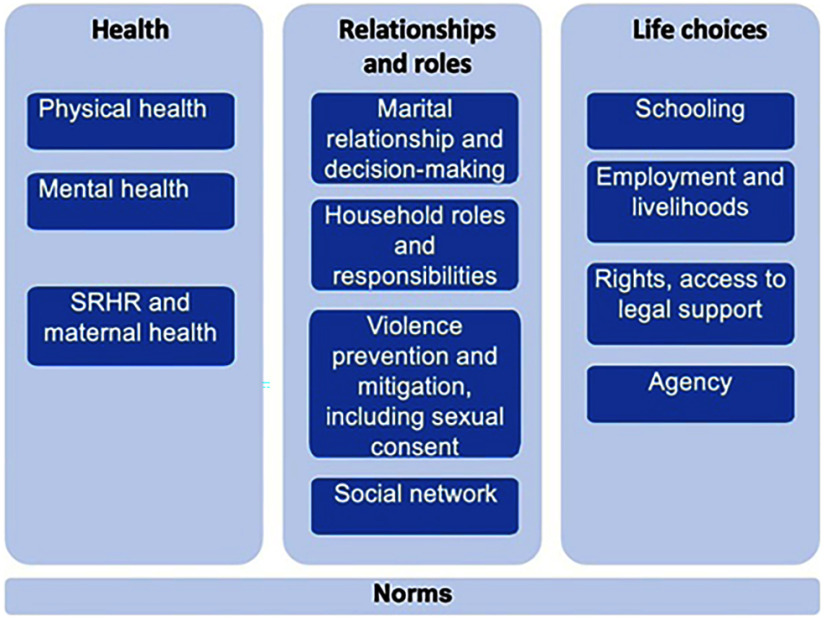
Conceptual Framework Guiding Analysis of Interventions to Respond to Needs of Married Adolescents

We mapped outcomes to a conceptual framework we developed before initial searches and then refined the framework as we reviewed the literature (Figure 2). This conceptual model captures the range of potential intervention areas that may support married adolescents, organized by 3 broad dimensions: health, relationships and roles, and life choices. Program activities addressing health include physical health, mental health, sexual and reproductive health (SRH), and maternal health (MH). Those activities included in the relationships and roles dimension address the marital relationship and decision-making, household roles, and violence including sexual coercion, and social networks to overcome social isolation and a lack of mobility. Program activities related to building girls’ life choices include efforts to increase or support schooling, build livelihoods, support an understanding of a person’s rights and legal support (for instances where a child might wish to challenge a marriage, face intimate partner violence, continue their schooling, separate, or seek a divorce, or seek child custody, for example), and build their agency or empowerment. Work to transform norms cuts across all 3 of these dimensions since a wide variety of normative expectations regarding the management of sexuality, transition to adulthood, deference to elders, future roles, and so on drive the pressure on girls, and sometimes boys, to marry and have children early.

## RESULTS

Table 2 summarizes the 29 intervention studies and their objectives mapped to the conceptual framework.^15^^–^^43^ Table 3 presents the intervention details, including evaluation design, quality, and key findings. Additional details on all studies are included in the Supplement Table S1. The data that generated this study’s findings are available on request from the authors.

**TABLE 2.** Interventions and Their Objectives Mapped to Conceptual Framework

**Conceptual Framework**	**Program Activity Areas Addressed by Interventions**
Health	Physical^15^^,^^20^
	Mental^37^
	Sexual and reproductive^15^^–^^36^^,^^42^^,^^43^
	Maternal^15^^–^^17^^,^^20^^,^^22^^,^^23^^,^^25^^–^^27^^,^^29^^,^^31^^–^^36^^,^^42^^,^^43^
Relationships and roles	Marital relationship and decision-making^15^^,^^18^^,^^19^^,^^21^^,^^23^^–^^26^^,^^29^^,^^33^^,^^36^
	Household roles and responsibilities^15^^,^^24^^,^^38^
	Violence prevention and mitigation, including sexual consent^15^^,^^24^^,^^27^^,^^38^^,^^39^^,^^40^
	Social network^20^^,^^22^^,^^23^^,^^26^^,^^30^^,^^39^
Life choices	Schooling^18^^,^^22^^,^^30^^,^^41^
	Employment and livelihoods^15^^,^^16^^,^^18^^,^^21^^,^^22^^,^^38^^,^^40^
	Rights, access to legal support^26^
	Agency^15^^,^^20^^,^^22^^,^^26^^,^^30^^,^^32^^,^^33^^,^^38^
Norms	Health and choice^20^^–^^22^^,^^24^^,^^26^^,^^31^^,^^33^^,^^34^
	Gender equity^23^^,^^24^^,^^26^^,^^36^^,^^38^
	Attitudes toward age at marriage^36^^,^^39^

**TABLE 3.** Characteristics of Included Studies Addressing the Needs of Married Girls and Results

**Study, Year, and Country**	**Study Objective and Interventions**	**Evaluation Design**	**Quality**	**Key Findings**
Erulkar and Tamrat,^15^ 2014Ethiopia	Provided girls with peer groups on SRH topics, financial literacy, safe motherhood, and self-esteem. Partner program provided training for husbands on partner communication, nonviolent and respectful relationships, caring for wives and children, and SRH topics.	Midline and endline population-based surveys	Low	Increased FP use, voluntary counseling and treatment, and husbands’ likelihood of helping with household, and mild increase in husbands’ likelihood of accompanying wife to clinic. However, if husbands were not also program participants, increased likelihood of being forced into sex and mild increase in wives’ likelihood of being beaten.
Handa et al.,^16^ 2015Kenya	Provided monthly unconditional cash transfers to poor females aged 12–24 years to measure impact on pregnancy and early marriage.	Cluster RCT	Medium	Decreased odds of pregnancy, especially among out-of-school girls.
Pathfinder International,^17^ 2015Burkina Faso	Trained CHWs to reach young married women and first-time parents with SRH information to increase contraceptive use and conducted small group discussions to engage community.	Qualitative monitoring data	Low	Increased contraceptive use.
Luseno et al.,^18^ 2017Zimbabwe	Provided school support (payment of school fees, uniforms, exercise books, and other supplies) to young orphaned women to examine effects on pathways to and experiences with marriage and use of maternal and child health services.	Quasi-experimental pre-and post-test with control; IDIs with sampled girls	High	Although quantitative results are not significant due to low sample size, women showed a decrease in positive attitudes toward SRH services and use of SRH services, as well as being less likely to use FP, get HIV testing, and immunize children. There was an increase in HIV positivity. Decreased happiness in marriage. Increase in +1 year education and food security. Equal perceptions of having resources compared to others.
Shattuck et al.,^19^ 2011Malawi	Provided peer-delivered (“male motivator”) educational and motivational intervention to examine effect on couples’ contraceptive uptake.	RCTIn-depth interviews	Medium	Increased contraceptive use, higher scale score for general communication with partners, and increased communication frequency with partners.
CREHPA,^20^ 2004Nepal	Held monthly peer group meetings for youth community action groups and mothers’ groups to improve access to and use of RH services and information by young married couples, ages 24 years and younger.	Quasi-experimental pre-post-test with 2 intervention arms (youth, mother’s groups) and 1 control	Medium	Increased awareness, knowledge, and acceptance of contraception, HIV/AIDS, and ANC, as well as increased knowledge of danger signs in pregnancy, labor, and delivery. Increased vitamin A consumption and deliveries assisted by traditional birth assistants in the youth community action group area.
Edmeades et al.,^21^ 2016Ethiopia	Group-based peer education meetings on SRH, economic empowerment, and conflict resolution and combination of those topics on contraceptive use and STI knowledge.	Quasi-experimental pre-post-test with 3 treatment arms plus controlNonrandom selection of eligible districts but random selection of implementation sites	High	Increased reported use of SRH services, basic knowledge of STIs, HIV testing, use of modern contraceptives, percentage of women who discussed FP with their husbands, as well as increased personal financial savings, intention to invest savings, and economic self-sufficiency.
Erulkar and Muthengi,^22^ 2009Ethiopia	A combination of peer group and safe social space, education support, and referrals to health services for girls to remain in school and community awareness to reduce the prevalence of child marriage in rural Ethiopia.	Quasi-experimental pre-post- with control, controlling for age, socioeconomic status, marital status, and years of schooling	Medium	Increased use of contraceptives; likelihood of talking with a friend about FP, condoms, HIV/AIDS, STIs; awareness of condoms; and knowledge that one can’t always tell if a man has an STI. Increase in discussion about violence in the community and problems in marriage and increase in new friendships. Increased enrollment in school and literacy.
Huda et al.,^23^ 2019Bangladesh	Peer groups of married adolescent girls aged 14–19 years in urban slums of Dhaka to discuss SRH topics combined with group leader training, community health volunteer SRH service promotion, FP material distribution, and counseling.	Quasi-experimental design using population-based surveys; IDIs and FGDs with wife participants and their husbands	Medium	Increase in odds of FP awareness and belief that FP is joint responsibility of husbands and wives.
Institute for Reproductive Health et al.,^24^ 2016Uganda	Used collective dialogue and action implemented by community leaders and mobilizers through community action groups, radio dramas, and village health teams to promote and sustain change related to social norms and attitudes toward gender, RH, and violence.	Quasi-experimental pre- and post- with control, propensity score analysis with difference-in-difference estimates	Medium	Increase in partner communication about FP, FP seeking behavior, use of FP, and intended future FP use. Increase in equitable partner-decision-making score and couple communication score. Increase in household role sharing score, men’s involvement in sharing of household roles, men involvement in at least 2 childcare tasks, as well as decrease in violent response to partner conflict.
Khan et al.,^25^ 2008India	Trained community workers to promote lactational amenorrhea and postpartum contraception among pregnant women with a parity of 0 or 1, distributed information and education materials, and conducted household visits.	Quasi-experimental pre-and post- with control, clustered by village, baseline and 2 endlines	Medium	Increased postpartum contraception use, postpartum lactational amenorrhea use, and knowledge of methods for spacing pregnancy.
Santhya and Haberland,^26^ 2007India	Provided health education and information to first-time parents on SRH topics, communication, and join decision-making, provide education to service providers on first-time parent needs, and form married girl peer groups to reduce isolation and increase agency.	Quasi-experimental study, 2 villages, baseline and endline surveys, no pre-assignment to treatment/controlIDIs at baseline.	Medium	Increase in RH knowledge (both Vadodara and Diamond Harbor villages)Diamond Harbor: increased number of women who had comprehensive antenatal check-ups, made delivery preparations, had a postpartum check-up, breastfed babies immediately after birth, and fed their babies colostrum.Vadodara: Increased number of women who had routine postpartum check-ups and increase in contraceptive use for delaying the first birth.Increased say in household decision-making, freedom of movement, friends in whom to confide, discussion of contraceptives with partners.Vadodara: Increase in egalitarian gender role attitudes and discussion of timing of first pregnancy with partners; Diamond Harbor: increase in openly disagree with husband.
Silverman et al.,^27^ 2019Niger	Used monthly household visits, peer group sessions, and community dialogues on healthy timing and spacing of pregnancy and SRH topics to increase use of modern spacing contraception among married adolescent girls (ages 13–19 years) and their husbands in the Dosso region.	Four-arm cluster RCT, difference in difference	High	Increase contraceptive use (household visit and combined arms only) and decreased intimate partner violence (small group sessions and combined arms only).
Subramanian et al.,^28^ 2018India	Conducted outreach to married young women and husbands of young women in Bihar on SRH and gender to delay the age at first birth by delaying the age at marriage, increase voluntary contraceptive use among young nulliparous married women, and to space second and subsequent births by at least 3 years.	Multiple quantitative population-based quasi-experimental evaluations from 3 project phases	Medium	Increased contraceptive use.
Undie et al.,^29^ 2014Kenya	Conducted a media campaign, CHW training, and distribution of information to promote the uptake of comprehensive RH (including FP) and HIV prevention services and information among married adolescent girls and expand access to these services among this population.	Pre- and post-intervention design without control, some measurement of exposure on select indicators	Low	Increased use of FP and postpartum use of implants and condoms, number of married girls decreased whose first ANC visit was at month 7-8 of pregnancy and who delivered babies at home and number of married girls increased who had 4 total ANC visits during pregnancy Increased proportion of girls whose husbands: provided transportation or transportation money to ANC services; provided money to pay for delivery; provided money for delivery services.
Mehra et al.,^30^ 2018India	Provided youth safe spaces, peer educator training, and community mobilization focusing on SRH information and education and assess effects on early marriage, early pregnancy, and school retention among young people in 2 states.	Cross-sectional post-test	Low	Delayed pregnancy.
Dyalchand et al.,^31^ 2021India	Behavior change communication through CHW visits to improve the RH of married adolescent girls and avert the adverse consequences of early motherhood	Quasi-experimental with pre- and post-test, difference-in-difference	Medium	Increased use of full ANC, consumption of at least 3 meals/day in the third trimester of pregnancy, safe and institutional delivery, use of postnatal care, and treatment of postnatal complications.
Engebretsen and Kaboré,^32^ 2011Burkina Faso	Mother educators provided information and support to married adolescents during their first pregnancy and birth and provided Vitamin A and iron supplements to those who were pregnant	Cross-sectional surveys of households and adolescent girls at baseline and endline	Low	Increased knowledge of obstetric fistula, use of SRH services, particularly delivery assistance, and knowledge of the minimum legal age of marriage.
Foundation for Research in Health Systems,^33^ 2006India	Through social mobilization and strengthening government RH services aimed to improve married adolescents’ SRH knowledge and to increase their access to and use of health services.	Quasi-experimental design pre- and post- with control, social mobilization arm, government services arm, and combined arm	Medium	Increased knowledge of maternal health and contraceptive side effects; increased postnatal check-ups, contraceptive acceptance, treatment of gynecological disorders, RTIs, and STIs; and increased husbands’ awareness of maternal health needs.
Mathur et al.,^34^ 2005Nepal	Used community mobilization and participatory approaches to improve services and outcomes for youth RH, focusing on youth aged 14–21 years.	Quasi-experimental pre-and post- with control; cross-sectional (not panel) data; IDIs and FGDs	Medium	Increased knowledge of serious problems during childbirth, use of ANC, and use of facilities for delivery.
Pande et al.,^35^ 2006India	Provided youth-friendly, accessible, affordable and effective diagnosis and treatment for RTIs and STIs among adolescents and young women and men through 2 different community-based approaches.	Quasi-experimental design with 2 study arms (community- based health aid, female doctor, control); FGDs with young men	Medium	Increased contraceptive use.
The ACQUIRE Project,^36^ 2008Nepal	Increased access to and use of RH information and services among married adolescents in Parsa and Dhanusha districts through peer education and network building.	Cross-sectional quasi-experimental with household surveys, no control; qualitative FGDs	Low	Increased use of ANC care and deliveries attended by a skilled birth attendant and decreased deliveries taking place at home. Increased communication and joint decision-making and awareness of needs and rights of married adolescents.
Malak et al.,^37^ 2021Jordan	Provided mental health counseling and evaluated depression, anxiety, and stress symptoms among adolescent married girls in Palestinian refugee camps.	Cross-sectional survey with convenience sample (no follow-up)	Low	Decreased depression, anxiety, and stress with previous trauma and father’s education; decreased anxiety and stress with age at marriage; and decreased stress with husband’s education and family income after marriage.
Falb et al.,^38^ 2015Côte d’Ivoire	Used group savings program and women’s discussion groups to promote gender equality, and violence prevention, improve livelihoods, and increase agency.	RCT	High	Decreased economic abuse for married girls and violence for unmarried girls.
Stark et al.,^39^ 2018 Ethiopia	Conducted adolescent girl life skills session in safe spaces and caregiver sessions to improve communication and raise awareness of GBV among refugee adolescent girls.	Two-arm, single-blinded, cluster RCT	High	No effect of intervention on reports of sexual violence, physical violence, emotional violence or transactional sex in the previous 12 months or on perceived feelings of safety.Increased perceptions of social support* *increased attitudes around ideal ages of marriage and motherhood.
Muthengi et al.,^40^ 2016Kenya	Intervention program aimed at building social, health, and economic assets for vulnerable married adolescent girls.	Cross-sectional secondary analysis of survey data coupled with content analysis of in-depth interviews	Low	Increased odds of experiencing physical violence for girls who worked compared to not-working, odds of experiencing physical violence for girls who worked with no regular saving compared to girls who did not work, and odds of experiencing physical violence for girls earning a higher (≥ median) income with no regular saving compared to not working.Decreased odds of experiencing violence for girls who reported their partner trusting them with money compared to those not having partner trust.
Walgwe et al.,^41^ 2016Kenya	Conducted interactive media campaign, advocacy, and policy dialogues to increase demand for secondary school education among teenage mothers.	Cross-sectional baseline and endline data	Low	Increased % out-of-school girls who reentered school.
Jacobs et al.,^42^ 2017Burkina Faso; Senegal	Used generic FP messaging through media campaigns to reach married adolescent women in West Africa to examine whether such messaging is associated with increased contraceptive use.	Cross-sectional, secondary analysis of DHS data using propensity score matching	Low	Increased odds of contraceptive knowledge and odds of intended future contraceptive use (Burkina Faso only).
KEM Hospital Research Centre,^43^ 2004India	Provided an integrated package of RH information, clinical referrals, and SRH couples counseling to married youth in a rural context.	Quasi-experimental pre- and post- without control plus quantitative and qualitative monitoring	Low	Increased couples’ knowledge of SRH (even if only 1 person participated).

Abbreviations: ANC, antenatal care; CHW, community health worker; DHS, Demographic and Health Survey; FGD, focus group discussion; FP, family planning; GBV, gender-based violence; IDI, in-depth interview; RCT, randomized controlled trial; RH, reproductive health; RTI, reproductive tract infection; SRH, sexual and reproductive health; STI, sexually transmitted infection.

### Methodological Quality and Study Duration

Five randomized control trials were represented among the studies (Table 3).^16^^,^^19^^,^^27^^,^^38^^,^^39^ The remaining studies used pre-test/post-test with comparison group design and/or quasi-experimental design or used cross-sectional data without comparison groups. Based on the results of the assessment of the methodological design and risk of bias assessment, most studies were of either high or moderate evaluation quality, but 7 were of low quality.^15^^,^^17^^,^^29^^,^^30^^,^^32^^,^^36^^,^^37^

Our analysis of the duration of each intervention by region in which the program was implemented shows that for the most part, studies were carried out in relatively short periods of time with 5 studies conducted within a study period of less than 18 months, 11 studies conducted over 18–35 months, and 11 studies conducted over 3 years or more (note that 2 studies did not report program duration) (Supplement Table S2). Seven of 13 of the longer-duration evaluations (those that lasted at least 3 years) were conducted in South Asia,^28^^,^^30^^,^^31^^,^^33^^–^^35^^,^^43^ with 2 conducted in East Africa^15^^,^^16^ and 1 each in West Africa^17^ and Southern Africa.^18^

### Geography and Target Population

Sixteen of the 29 studies were carried out in sub-Saharan Africa, including 3 in Burkina Faso,^17^^,^^32^^,^^42^ 4 in Ethiopia,^15^^,^^21^^,^^22^^,^^39^ 4 in Kenya,^16^^,^^29^^,^^40^^,^^41^ and 1 each in Côte d’Ivoire,^38^ Malawi,^19^ Niger,^27^ Senegal,^42^ Uganda,^24^ and Zimbabwe.^18^ Eleven studies were conducted in South Asia with the highest representation in India of 8 studies,^25^^,^^26^^,^^28^^,^^30^^,^^31^^,^^33^^,^^35^^,^^43^ followed by 3 in Nepal^20^^,^^34^^,^^36^ and 1 in Bangladesh.^23^ Only 1 program was identified from the Middle East and North Africa (Jordan).^37^ We identified no studies from Latin America and the Caribbean, nor from Europe, Central Asia, or North America.

Fifteen programs included only currently married girls, and 13 programs included both married and unmarried girls (Table 3). Only 1 program targeted males,^19^ although 15 studies engaged boys and men in some capacity as girls’ husbands and partners. There were 16 programs involving community members (religious and traditional leaders, health care providers) and/or extended family (mothers-in-law, caregivers, co-wives). Three studies included unmarried girls such as those who were ever-married (perhaps married previously but not currently), 3 who were cohabiting, and 1 pregnant and parenting. Programs often included different age groups, including 15 studies on girls aged younger than 15 years.

Our analysis of study findings draws from Table 3 and Supplement Table S1, which describes the components of the interventions, and displays detailed results for the various outcomes. Our narrative presents the analysis by areas of the conceptual framework.

### Intervention Category in Conceptual Framework

Twenty-five of the 29 studies intended to measure health outcomes; and 24 of those focused on SRH and MH.^15^^–^^36^^,^^42^^,^^43^ There were only 2 studies on general physical health^15^^,^^20^ and 1 on mental health.^37^ Following health, the next most represented category of interventions was relationships and roles with 18 of 29 studies. Within relationships and roles, 11 of the 18 studies addressed the marital relationship, 6 studies addressed violence prevention ^15^^,^^24^^,^^27^^,^^38^^–^^40^ and 6 on girls’ social networks.^20^^,^^22^^,^^23^^,^^26^^,^^30^^,^^39^ Only 3 of 18 studies addressed household roles and responsibilities.^15^^,^^24^^,^^38^ Lastly, of the 3 broad categories of interventions represented in the conceptual framework, the fewest studies (13 of 29) focused on building life choices, with 8 of 13 on agency,^15^^,^^20^^,^^22^^,^^26^^,^^30^^,^^32^^,^^33^^,^^38^ the most common subcategory, followed by 7 on livelihoods,^15^^,^^16^^,^^18^^,^^21^^,^^22^^,^^38^^,^^40^ 4 on schooling,^18^^,^^22^^,^^30^^,^^41^ and 1 on rights and access to legal support.^26^

The majority of the studies intended to measure health outcomes focused on SRH and maternal health.

Underpinning the domains of health, relationships and roles, and development of life choices, 12 different programs aimed to measure norms among their outcomes. These efforts focused on improving community awareness, support, and/or advocacy of the SRH and MH needs of married adolescents (10) as well as promoting gender-equitable attitudes and behaviors among youth (6). It is worth noting that 22 of the 29 studies focused on multiple intervention approaches. In particular, 11 studies targeting SRH overlapped with marital relationship, 10 with norms, 7 with agency, and 5 with social networks. In other words, most studies measuring non-SRH outcomes also measured SRH.

#### Health

Among the 24 studies that aimed to improve girls’ SRH and/or MH, 3 critical intervention points emerged: efforts to increase contraceptive use, efforts to delay pregnancy, and efforts to improve quality, access, or use of SRH and MH services. Thirteen studies^15^^,^^17^^,^^19^^–^^29^ documented increased contraceptive use through activities, such as peer group education sessions,^15^^,^^17^^,^^19^^–^^22^^,^^24^^,^^26^^–^^28^ community media, such as radio, social media, or community theater,^17^^,^^24^^,^^25^^,^^29^ and services provided by community health workers (CHWs) (like household visits or referrals/escorts to health services)^17^^,^^23^^,^^25^^–^^29^ (Table 3). Eleven of these studies also involved husbands or partners.

Three studies reported delays in pregnancy.^16^^,^^25^^,^^30^ The pairing of an educational campaign and community worker counseling on healthy timing and spacing of pregnancies in India contributed to a decrease in the incidence of pregnancy at 9 months postpartum (using a quasi-experimental pre-and post-evaluation design with control).^25^ In addition, both the unconditional cash transfers in Handa et al.^16^ and the combination of sexual and reproductive health and rights (SRHR) life skills-based peer education sessions, community posters, and meetings with local leaders, advocacy and awareness campaigns, and collective action in Mehra et al.^30^ led to lower likelihood of pregnancy (Table 3).

Fourteen studies documented improvements in quality, access to, or use of SRH and MH services, including the use of health information.^15^^,^^17^^,^^20^^,^^21^^,^^24^^–^^26^^,^^29^^,^^31^^–^^36^ Ten programs worked to improve the quality of services, advocating for accessible youth-friendly SRH services,^17^^,^^20^^,^^24^^,^^32^^,^^35^^,^^36^ providing medical equipment to facilities and birth attendants,^26^^,^^36^ and looking to communities to monitor service provision.^25^^,^^31^^,^^33^ Measurement of the quality of services was inconsistent if it was captured at all; Center for Research on Environment Health and Population Activities (CREHPA)^20^ and Dyalchand et al.^31^ measured service use and knowledge rather than quality.

Five interventions increased married girls’ seeking of antenatal care (ANC)^20^^,^^26^^,^^29^^,^^31^^,^^36^ using a diverse set of activities including organizing local advocacy groups, community monitoring of MH service providers, and engaging married girls’ parents. For example, CREHPA^20^ organized young community action groups to advocate locally for youth-friendly reproductive health services, and the proportion of young mothers in the community who attended 4 or more ANC visits more than doubled from 24.8% to 52.9% (*P*<.01), with vitamin A consumption rising as well. By contrast, Dyalchand et al.^31^ sent CHWs to refer married girls for SRH and MH services while implementing community-based monitoring of service provision, and their ANC service use also increased, by 47.8 percentage points (from 8.2% to 56.1%) over 17.2% in the control. Both CREHPA and Dyalchand et al. increased ANC use and the proportion of births assisted by a professional.

Among other health outcomes, 4 studies measured the use of postnatal services,^26^^,^^31^^,^^33^^,^^34^ with the Foundation for Research in Health Systems^33^ finding that social mobilization was most effective in boosting postnatal visits. Four studies assessed outcomes related to reproductive tract infections or sexually transmitted infections.^15^^,^^21^^,^^33^^,^^35^ Four studies reported outcomes related to the use of SRH and MH information that did not require the use of formal services.^20^^,^^26^^,^^31^^,^^32^ For example, Dyalchand et al. reported on a program in India that used behavior change communication in household visits from CHWs to influence an increase in the consumption of at least 3 meals a day among pregnant married girls in their third trimester from 56% at baseline to 82% at endline, a significant rise compared to the control group (*P*<.01).^31^

Beyond SRH, only 2 studies^15^^,^^20^ included an activity related to broader physical health, and only 1 study^37^ focused on mental health. The interventions that offered physical health activities did not measure any physical health outcomes. These interventions used peer education groups of married girls or their husbands to discuss the consumption of alcohol and/or drugs, among other topics mainly focused on SRH. Similarly, the 1 study that addressed married girls’ mental health provided counseling services for those who needed them, but the lack of a post-test means the impact of these services was not evaluated, and no associated changes in depressive, anxiety, or stress symptoms among married girls were reported.

#### Relationships and Roles

Eighteen of 29 interventions aimed to affect married girls’ relationships and roles through activities addressing marital relationship dynamics (partner communication and decision-making [9] or enhanced relationship satisfaction [1]), household division of labor (3), violence prevention (6), and broader social networks (6). Nine of 18 studies viewed girls’ relationships and roles through an SRH lens.

Eighteen of 29 interventions aimed to affect married girls’ relationships and roles through activities addressing marital relationship dynamics, household division of labor, violence prevention, and broader social networks.

Reflective of the literature on married girls as a whole, interventions that attempted to influence child brides’ marital dynamics often adopted an SRH lens.^15^^,^^23^^,^^29^^,^^33^^,^^36^ The most common activities were peer-based education and counseling for girls themselves,^21^^,^^26^^,^^36^ in contrast to Khan’s sensitization campaigns and group counseling for husbands and mothers-in-law^25^ and ACQUIRE Project’s individual engagements through household visits.^36^ Nevertheless, each increased the intramarital discussion of either family planning (FP) or contraceptive use.

Undie et al. reported on a pre-and-post evaluation of a bundled intervention combining an interactive radio campaign, Facebook discussion group, and household visits by CHWs that showed increases in the number of husbands who provided transportation/transportation money to go for ANC services 53%–65% (*P*=.001), money to plan for the delivery 69%–79% (*P*=.003), and money to pay for delivery services 62%–60% (*P*=.021).^29^

Six articles measured broader partner communication and decision-making outside of SRH: 3 on partner communication,^19^^,^^24^^,^^26^ 2 on sharing housework and childcare,^15^^,^^24^ and 1 on marital happiness.^18^ Institute for Reproductive Health’s (IRH) radio drama campaigns combined with community health volunteer gender reflections,^24^ as well as the peer-delivered gender education for men in both Shattuck et al.^19^ and Santhya and Haberland,^26^ demonstrated increases in partner communication quality scales (all 3), equitable gender role scales (IRH and Santhya and Haberland), frequency of partner communication (Shattuck et al.), and instances of openly contradicting husbands (Santhya and Haberland).

On sharing housework and childcare, IRH^24^ and Erulkar and Tamrat^15^ reported a greater endline likelihood that a husband would contribute to housework. The intervention in Ethiopia from Erulkar and Tamrat trained husbands on partner communication, nonviolent and respectful relationships, caring for wives and children, domestic violence, and sexual violence, reporting 47.7% and 25.9% gaps over the control in husbands’ contributions to housework with and without husband participation in the program, respectively.

Luseno et al., the lone study investigating happiness in marriage, explored whether orphaned, married girls benefitted from paid school fees and other costs, such as uniforms and a female focal staff member to support them in schools.^18^ They retrofitted a sample of 35 such girls from a prior randomized controlled trial on the effect of this school support on HIV testing. Though their results are not significant, they were the only study to measure girls’ perceptions of happiness within marriage and their regrets about getting married, critical dimensions of marital well-being that are otherwise neglected by the literature.

Six programs aimed to reduce intimate partner violence (including physical violence, sexual violence, and economic abuse), with peer-group (5 studies) and education-related activities on non-violence and gender roles (4 studies) being the most common.^15^^,^^24^^,^^27^^,^^38^^–^^40^ Only 1 intervention offered household visits and referrals to health services,^24^ and no studies involved counseling. Their results were mixed and seemingly dependent on the target populations. In Falb et al., gender discussion groups significantly reduced physical, emotional, and sexual violence by half only for non-married girls, and not significantly for married girls, while reducing economic violence by two-thirds for both.^38^ Strikingly, in cross-sectional endline data, Erulkar and Tamrat observed a greater likelihood of physical and sexual violence (both 1.6 times greater) if wives participated in the program discussion groups without their husbands, and no difference between both wife and husband attending and neither attending.^15^ Taken together, these 2 studies may suggest that girls’ social environments and norms are more constrictive and less malleable to outside interference if they are married, unless husbands are engaged as well.

While many interventions engaged married girls’ social networks, only 6 interventions explicitly aimed to build and promote friendships among married girls and peers, and only 3 measured outcomes related to friendship/mentorship.^22^^,^^26^^,^^39^ For example, Erulkar and Muthengi^22^ and Stark,^39^ both of which included married and unmarried girls, found increased access to safe spaces to build friendships, plus the presence of adult female mentors increased self-report of having a friend one’s own age (Erulkar and Muthengi: 15% difference-in-difference; Stark: 1.71 times more likely than control).

#### Life Choices

Of the 13 studies that implemented activities related to expanding girls’ life choices, outcomes were distributed between schooling (4), employment and livelihoods (7), rights and access to legal support (1), and agency (8). In Kenya, a combination of radio messages and advocacy through policy dialogues encouraged school retention and re-entry for pregnant and parenting students (one-third of whom were married), leading to an increase in the proportion of out-of-school girls who re-entered school from 10% at baseline to 16% at endline.^41^ The only intervention specific to married girls was a school support program in Zimbabwe that reported an additional year in school for intervention over control participants, but these results are not significant due to the small sample size.^18^

Employment and livelihood outcomes were identified in 7 studies.^15^^,^^16^^,^^18^^,^^21^^,^^22^^,^^38^^,^^40^ These programs generally attempted to improve financial literacy or to provide financial support through access to income-generating activities or cash transfers. However, these outcomes were seldom measured; 1 notable exception was found in the significant increase (from 27% at baseline to 54% at endline; *P*<.1) among ever-married adolescent girls in Ethiopia who intended to invest their personal savings after participation in a combined SRH-economic empowerment training program.^21^ Among the studies that provided financial support to participants, 2 implemented village savings and loan association models through which women could pool their resources, request loans, and receive interest on loans repaid by other group members;^21^^,^^38^ 1 provided unconditional monthly cash transfers to households with orphans and vulnerable children;^16^ and 1 paid for school costs.^18^ The Reduction of Gender-Based Violence Against Women program in Côte d’Ivoire used the group savings model and measured significantly lower odds of child bride participants reporting economic abuse in the past year (odds ratio=0.33; *P*=.02) compared to a control group.^38^

Only 1 study, Santhya and Haberland, conducted an activity in rights and access to legal support, including basic reference to legal literacy in the context of peer education groups for married girls.^26^ While the program has outcomes related to marital relationship and shared decision-making, the authors did not measure knowledge of or access to legal support.

Agency, or the capacity to define one’s goals and act on them,^44^ was addressed through programs that sought to amplify married girls’ voices through youth-to-youth communication,^15^^,^^20^^,^^22^^,^^33^ build married girls’ self-esteem,^15^ and strengthen their life skills through vocational and human rights training.^26^^,^^32^ These studies generally measured married girls’ agency in terms of their marital relationships, FP decision-making, or violence reduction. Mobility, another important facet of agency, captures expectations regarding time use, friendships outside the household, and the regulation and control of young women’s sexuality. The First Time Parents Program in India affected married girls’ mobility and social isolation through girls’ group meetings and activities and whether they had a friend in whom to confide.^26^

### Norms

Underpinning the domains of health, relationships, roles, and life choices are the diverse norms that tend to constrain and disadvantage young married women, yet many evaluations choose not to measure changes in norms. Fourteen different programs addressed norm change by improving community awareness, support, and/or advocacy for the SRH and MH needs of married adolescents (10) and promoting gender-equitable attitudes and behaviors among youth (6). Three areas of focus emerged, with 8 studies addressing norms regarding health and choice;^20^^–^^22^^,^^24^^,^^26^^,^^31^^,^^33^^,^^34^ 5 studies addressing promoting gender-equitable attitudes;^23^^,^^24^^,^^26^^,^^36^^,^^38^ and 2 interventions addressing attitudes toward age at marriage or motherhood.^36^^,^^39^

Fourteen programs addressed norm change by improving community awareness, support, and/or advocacy for the SRH and MH needs of married adolescents and promoting gender-equitable attitudes and behaviors among youth.

Norms change focused on the awareness of and support for married girls among community gatekeepers, mothers-in-law, husbands, and health care providers, all of whom can constrain girls’ behavior. Of the 8 intervention studies that addressed these norms, IRH implemented community mobilization and radio dramas (aimed at changing norms on SRH and violence prevention).^24^ They documented more equitable partner decision-making relative to the control (9%), partner communication about FP (12%), FP-seeking behavior (16%), current FP use (10%), and intended future use (10%), and reduced violent responses to partner conflicts by 16% (results all significant at the *P*<.05 level).

Five interventions were evaluated for their efforts to promote gender-equitable attitudes between members of the couple, in the community, and among providers. One intervention with married adolescent girls clubs in Bangladesh described marriage registrars counseling couples together on topics including the role of men in FP, leading to odds ratios of 3.4 among participants for the belief that FP is the joint responsibility of both husbands and wives compared to the control area.^23^ Activities to shift norms at the community or health provider level also yielded results: newly married and newly parenting participants experienced increases in equitable partner decision-making (9%), household role-sharing (7%), couple communication (12%), men’s involvement in sharing of household roles (17%), and men’s involvement in at least 2 childcare tasks (10%) (all results significant at the *P*<.05 level).^24^

Of 2 interventions working to shift norms regarding motherhood and age at marriage, the program in Ethiopia described by Stark et al. supplemented weekly discussion sessions for adolescent girls, safe spaces, and a mentor with 8 monthly discussion groups for enrolled girls’ caregivers and resulted in an increased proportion of girls holding the view that marriage and motherhood should occur after the age of 18 years.^39^

## DISCUSSION

This review is the first attempt to synthesize the global evidence on interventions to support individuals married as children. Much is known about the consequences of child marriage, yet that understanding has not translated into action in support of those who have undergone this transition. We identified only 29 studies that met our criteria, a small number relative to the literature on interventions to prevent child marriage. Our analysis of study duration by subregion (Supplement Table S2) showed a remarkable geographic concentration of these interventions in South Asia and East Africa, with somewhat fewer in West and Southern Africa and a single program in the Middle East and North Africa region. Not a single study surfaced on Latin America and the Caribbean, North America, East Asia, or Europe and Central Asia. This may partly reflect our omission of research on informal unions but also exposes a lack of attention to married girls in these regions. And boys, by virtue of the fact that they marry as children at lower rates and may experience fewer harmful consequences, are virtually invisible throughout the literature.

Although much is known about the consequences of child marriage, that understanding has not translated into action in support of those who have undergone this transition.

The conceptual framework that guided our analysis captures the many domains of girls’ lives in which support and services are needed. Of 29 studies, 25 focus on SRHR and maternal health, leaving many other categories of well-being understudied. Donor-driven investment in SRHR and an interest in adolescent childbearing have likely played a role in shaping this research focus. Regardless, the result is research neglect in education and livelihoods, mental health, marital relationships, household roles, and legal rights and law—all areas that the literature on the consequences of child marriage has highlighted.

The broader literature highlights girls’ vulnerabilities after marriage to violence (e.g., social isolation, powerlessness, and a lack of access to educational opportunities).^45^^,^^46^ Yet our review shows that in education and work, married girls’ human capital is not being invested in. The challenges they face in their households and marital relationships are not being addressed, nor are the demonstrated links between low socioeconomic status and poor mental health outcomes^3^ and between low socioeconomic status and early marriage.^47^ Additionally, our review uncovered gaps in programming focused on providing married girls access to legal aid and support, including in the context of protection from violence and support for those wishing to leave marriage. Only 1 study referenced activities to address married girls’ legal rights, and it did not measure related outcomes.^26^ This affirms research by Roberts that “the emphasis on adolescent pregnancy prevention has been accompanied by lack of insight into the experiences of adolescent parents and their children.”^48^

We identified several gaps in programming activities. First, 5 of 6 violence-prevention interventions employed peer group formation and/or education sessions, and only the GREAT Project used referrals to health services or household visits.^24^ Yet we know from gender-based violence prevention literature that referrals and household visits are essential components of programs to prevent violence.^49^ Second, of the 7 interventions with an economic livelihoods component, only 2 included a cash or asset transfer, even though interventions that support girls’ schooling through cash or in-kind transfers are most often successful in preventing child marriage.^13^ The literature indicates that once girls are married, few interventions attempt to mitigate the harms of child marriage across these domains.^9^ These findings indicate that child marriage is treated as the end of opportunity, “as if once married, the options for exercising agency are non-existent.”^44^

In a related shortcoming of the field, many programs address conceptual framework outcomes that they ultimately do not measure, particularly in health care quality, relationship dynamics, and norms. The norms-related intervention studies we identified typically measure attitudes as proxies for norms, and they prioritize harnessing norm change in service of other outcomes rather than measuring shifts in norms themselves.

Lastly, there is an acute inattention to husbands—boys and men—in the context of child marriage. Only 1 intervention (Shattuck et al.^19^) targeted men as the principal program participants. The outcomes for men that are measured often reflect limited contributions (e.g., to provide money or transportation to FP services and to agree rhetorically that FP is joint responsibility). Yet multiple studies found that few husbands actually accompany their wives to clinics,^15^^,^^33^ suggesting the need for more creative and robust measures of husbands’ active participation in health.

Our analysis indicates that for most outcome categories for married girls, no single program approach emerges as the most effective. For example, 13 studies demonstrated improved rates of contraceptive use, yet their respective programs conducted activities as diverse as peer-based education, household visits, health care referrals, and media campaigns. Eight studies with a similar mix of program activities each reported improved marital communication. Interestingly, however, at least 3 studies concluded that single intervention approaches produced stronger effects than multicomponent programming, which they attributed to the more focused effort. For example, Edmeades et al.^21^ compared outcomes for study arms with an SRH intervention only, an economic empowerment intervention only, and a combined intervention and found that 4 of 5 SRH outcomes were improved by participation in either single intervention arm, above the combined arm. Single intervention arms also outperformed combined intervention arms in both Subramanian et al.^28^ and the Foundation for Research in Health Systems study.^33^ It seems that robust and well-funded programming is more important than the specific program approach.

Our analysis indicates that for most outcome categories for married girls, no single program approach emerges as the most effective.

In similar arm-by-arm comparisons, 3 studies found that community mobilization outperformed other programming activities in supporting married girls.^20^^,^^33^^,^^34^ For example, Mathur et al. found that a “traditional” SRH approach outperformed in increasing knowledge of ANC services and use of facilities for delivery, but community mobilization outperformed in the use of those services.^34^ Because married girls face more social and cultural constraints than unmarried girls, it may be more important to engage their broader social networks.

Indeed, the mobilization of married girls’ families and communities is not often measured, but it is logically central to these interventions’ mechanisms of change. Most interventions organized married girls into peer groups, and many also relied on household visits from community workers. These types of activities, in addition to explicit community mobilization, strengthened married girls’ social networks (e.g., Edmeades et al.,^21^ Erulkar and Muthengi^22^) in service of other outcomes of interest.

Our review also finds that 2 key gatekeepers within married girls’ social networks are their husbands and mothers-in-law, who may constrain girls’ behavior even when girls’ own knowledge and attitudes have been improved.^17^ Falb et al. found that married girls were unaffected by an intervention that reduced rates of violence for unmarried girls.^38^ Once married, girls may be so constrained by these gatekeepers that their own changed knowledge and attitudes may not affect behavioral outcomes. Practitioners should account for the distinct barriers faced by married and unmarried girls when planning interventions designed to help both.

Finally, we highlight some common methodological shortcomings of the evidence on married adolescents. Most studies in this review are quasi-experiments, some lack control groups, and some are purely observational. For example, Malak et al., the only study exploring girls’ mental health, presents only observational baseline data.^37^ Some sample sizes are small (e.g., Luseno et al.^18^ at 35) across treatment and control. Moreover, given the wide variation in girls’ cultural, economic, and political contexts, even a fully randomized experiment of a program may yield results that are valid but not informative for a similar program in the next province.

### Limitations

One of the limitations of our review was that we only included interventions where findings were presented by marital status. While we applied this criterion so that the effect of the intervention on married adolescents, specifically, could be discerned, this led to the exclusion of select interventions that included married adolescents in their sample but did not report the outcomes for those married adolescents. Additionally, the research team excluded “adolescent mothers” as an additional search term to focus on child marriage. Consequently, our scoping excluded potentially relevant studies in North America and Europe and perhaps other regions as well. Additionally, the review was limited by its inclusion of English-only publications.

Many programs also tend to lack reporting on details associated with implementation or evaluation, with few studies providing details on attrition rates, for example. Insight into the feasibility of replicating or scaling up studies is also limited, given how infrequently studies conduct cost-benefit analyses.

It is also worth noting that these results are subject to 2 types of bias: publication bias across studies and sampling bias within studies. Positive findings are more likely to be published than null findings, perhaps unduly strengthening potential conclusions on how effectively programs support married girls overall.

## CONCLUSIONS AND CALL TO ACTION

Our review is the first to assess the evidence on interventions for individuals married as children. We find that the child marriage field is largely focused on prevention, at the neglect of a robust response. Married girls are allowed to vanish into their marriages with little attention to their needs. What is more, the programs that exist are of interest but occur mostly at small scale, are geographically concentrated, and focus on limited sectors. Boys marrying as children is such a neglected topic that there are virtually no programs for them.

Our findings point to a number of specific interventions. Greater investment in targeted programming for married girls is needed to address the dearth of evidence on effective approaches. While the issue of child marriage has been well defined, funding should increasingly focus on testing scalable interventions tailored to local child marriage practices, with robust data collection on program implementation, monitoring, and evaluation. Lessons can be learned by the child marriage field from other sectors, including the focus of the gender-based violence sector on prevention, risk mitigation, and response. This requires the timely identification, support, and referral of married children, strengthening the delivery of programs and services, and addressing the barriers to accessing support. The focus on whether a girl marries before or after age 18 years precludes mitigation in some instances (i.e., delay may be overlooked or go unmeasured if the person still marries as a child).

We strongly recommend testing more educational and economic interventions for married girls in countries with a high child marriage burden. Education equips individuals with essential knowledge, practical skills, and broader perspectives, enabling them to overcome challenges, actively engage in their communities, and pursue fulfilling personal and professional paths. This is a prime area of inconsistency between the response to the needs of unmarried and married girls, given the importance of these investments for prevention.

We strongly recommend testing more educational and economic interventions for married girls in countries with a high child marriage burden.

The reduced impact of interventions among married girls compared to unmarried girls strongly suggests the need to bolster their resources and opportunities and to strengthen their position within their marriages and broader family settings. Policies that sustain connection with married girls and link them to advance their schooling and build their social and economic capital are sorely needed.

Programmatically, some of what has been done for adolescent mothers in the United States may be relevant to global work on child marriage and efforts to improve the lives of married girls. The emphasis in North America has been on the transition signaled by childbearing in adolescence rather than marriage in adolescence. Yet there is interesting experience, particularly in providing supports for schooling, home visits, and mentoring. A shortcoming of intervention research in both the United States and in low- and middle-income countries has been that it tends not to reference partners or husbands, even when the focus is on preventing a second pregnancy.^50^ This reinforces the tendency to overlook relationship dynamics, partners, boys, men, power, sex, and so on, consistent with the broader neglect of boys we have noted elsewhere.

Given the attention accorded to norms in the background sections of quite a few of the studies, we also recommend shifting from solely measuring individual-level gender attitudes and beliefs towards the measurement of norms (what “others” believe and do) with explicit consideration of reference groups (e.g., perceptions of friends, family, and community). Understanding how norms vary across groups will also be useful in planning interventions, as it can guide investment to groups where norms are most entrenched or catalyze efforts for larger normative change in areas where norms are weaker or more contested.^51^ Longer intervention timeframes and more intensive targeting of influential family and community members will be needed to affect cultural beliefs and behaviors that negatively impact youth reproductive health decision-making and outcomes. We also recommend more attention to measuring social and legal support for individuals married as children.

The normative attitudes and assumptions of researchers, program designers, and donors toward marriage influence the field’s approach to working with married girls. Little attention is paid to married girls, as though it were too late to reach them. Limited effort is made to address relationship dynamics and other conditions within marriage other than SRH, perpetuating a view that marriage is private. More broadly, we must acknowledge the diversity in reasons for and conditions of child marriage, factors that shape the relational and situational constraints of a marriage, and its impact on marital life for a girl.

More attention is needed to describe and measure the consequences of early marriage for boys. Until we know more about their experiences within marriage, the field will be ill-equipped to develop any programs to support them.

Support for individuals married as children represents a blind spot in international human rights. The limitations of programs designed to address the needs of individuals married as children reflect tensions around defining age, the ability to consent, the vulnerability of children, and children’s need for protection from adults versus the need for adults to provide protection. Agency in child marriage is commonly considered important before marriage to resist marriage (and fails to account for adolescents choosing to be married or agency that may be exercised after child marriage).^42^ The lack of evidence on responses to married girls points to a need to pay greater attention to the issue of agency and to the participation of married girls and boys in the design, development, and evaluation of programs.

## Notes

**Peer Reviewed**

**First Published Online:** July 25, 2024.

**Cite this** article **as**: Siddiqi M, Greene ME, Stoppel A, Allegar C. Interventions to address the health and well-being of married adolescents: a systematic review. *Glob Health Sci Pract*. 2024;12(4):e2300425. https://doi.org/10.9745/GHSP-D-23-00425
